# Different Analysis of *β*-Cell Dysfunction as Fasting Glucose Progresses in Obese and Nonobese Newly Diagnosed Type 2 Diabetic Patients

**DOI:** 10.1155/2019/6053604

**Published:** 2019-10-28

**Authors:** Yan Duan, Xiaomeng Sun, Jia Liu, Jing Fu, Guang Wang

**Affiliations:** Department of Endocrinology, Beijing Chao-Yang Hospital, Capital Medical University, Beijing 100020, China

## Abstract

**Aims/Introduction:**

This study is aimed at (1) investigating the change of *β*-cell dysfunction as baseline fasting glucose progresses in newly diagnosed patients with T2DM and (2) finding whether body mass index (BMI) has different degrees of impact on insulin secretion as baseline fasting glucose progresses.

**Materials and Methods:**

661 patients with newly diagnosed T2DM were enrolled in the present study. A 75 g oral glucose tolerance test was used to calculate HOMA-*β*, HOMA-IR, early-phase insulin secretion index (EISI, calculated as *Δ*I_30_/*Δ*G_30_), and area under the insulin releasing curve (AUC_I0-180_). Patients were divided into low, medium, and high FBG groups. Each group was further divided into lean, overweight, and obese subgroups according to BMI.

**Results:**

A decrease of EISI and HOMA-*β* and an increase of HOMA-IR were shown among different FBG groups significantly. In the medium FBG group, AUC_I0-180_, EISI, HOMA-*β*, and HOMA-IR in obese patients were higher than those in lean and overweight patients. In the low and high FBG groups, AUC_I0-180_, HOMA-*β*, and HOMA-IR in obese patients were higher than those in other subgroups. BMI was positively associated with high EISI in the medium FBG group but failed to yield a significant association with EISI in the low and high FBG groups.

**Conclusions:**

During the progression of baseline FBG, *β*-cell dysfunction and insulin resistance worsened. As FBG increased, increased BMI had a positive influence on *β*-cell dysfunction in all FBG groups. The independent factors that correlated to EISI differed with the increasing of baseline FBG.

## 1. Introduction

Type 2 diabetes mellitus (T2DM) has become a major global public health concern, as its serious complications and morbidity rate increase worldwide. Both insulin secretion defect and insulin resistance are major mechanisms of T2DM. Islet *β*-cell dysfunction occurs at the early stage of the development of diabetes. A previous study found that the early and late phases of insulin secretion were decreased as fasting blood glucose (FBG) increased in nondiabetic people. When FBG is at 5.6 to 6.1 mmol/L, the early phase of insulin secretion has decreased to 50% of the maximum [1].

Early-phase insulin secretion plays an important role in the development of diabetes. It reduces both postprandial blood glucose and the increase of insulin by inhibiting the production and output of hepatic glycogen and the secretion of glucagon. It also decreases the level of postprandial free fatty acids (FFAs) by restricting FFA release into the blood [2, 3]. In prediabetes, Weyer et al. found that insulin secretion defects have occurred, and the peak value of early-phase insulin secretion can predict the occurrence of impaired glucose tolerance (IGT) and T2DM [4].

Patients with T2DM are likely to combine overweight and obesity. Obesity causes insulin resistance and islet *β*-cell dysfunction. A recent study showed that waist-to-hip ratio was negatively correlated with both the early and late phases of insulin secretion among obese patients [5]. It is vital to correctly evaluate the pancreatic function of patients with diabetes to protect the residual pancreatic function through appropriate therapy, whereas whether BMI has different degrees of impact on insulin secretion in different levels of blood glucose as the FBG increases is still unclear.

## 2. Materials and Methods

### 2.1. Design and Participants

The present study was a cross-sectional study of patients with newly diagnosed T2DM in the endocrine outpatient department of Chao-Yang Hospital. These patients were diagnosed with T2DM based on the 1999 World Health Organization (WHO) diagnosis criteria. Those who had not received any oral hypoglycemic drug and those who were treated for a short period of time but had discontinued at least 3 months before the enrollment were included in the study. The exclusion criteria were as follows: patients with a history of acute myocardial infarction, unstable angina, renal function impairment, liver function impairment, hematological diseases, chronic hypoxic diseases (emphysema and cor pulmonale), and infectious disease. Patients who did not complete the oral glucose tolerance test (OGTT) and had EISI ≤ 0 were also excluded. A total of 661 patients (389 male and 272 female) aged between 30 and 70 years were enrolled in the current study.

### 2.2. Measurements

Height, weight, waist circumference, systolic blood pressure (SBP), and diastolic blood pressure (DBP) were measured. BMI was calculated as weight (kg)/height (m^2^). Laboratory indices included triglycerides (TG), total cholesterol (TC), low-density lipoprotein cholesterol (LDL-C), high-density lipoprotein cholesterol (HDL-C), and hemoglobin A1c (HbA1c). A 75 g OGTT was used to evaluate insulin secretion function and insulin resistance among the patients. Blood glucose and insulin concentration were tested at 0, 30, 120, and 180 minutes through OGTT.

Insulin secretion function indices were calculated using OGTT results as follows:

- Fasting insulin (FIN) (mU/L) = 0-minute blood insulin concentration
- FBG (mmol/L) = 0-minute blood glucose
- PBG (mmol/L) = 120-minute blood glucose
- Homeostasis model assessment of *β*-cell function (HOMA‐*β*) = 20∗FIN/(FBG − 3.5)
- Homeostasis model assessment of insulin resistance (HOMA‐IR) = FBG∗FINS/22.5
- The measure of early-phase insulin secretion index (EISI): ΔI_30_/ΔG_30_ = (INS_30 min_ − FINS)/(Glu_30 min_ − FPG)
- The measure of total insulin secretion: AUC_I0−180_ = 0.5∗(FINS + INS_30 min_)∗30 + 0.5∗(INS_30 min_ + INS_120 min_)∗90 + 0.5∗(INS_120 min_ + INS_180 min_)∗60

### 2.3. Distribution of Patients

Based on the tertiles of FBG, patients were categorized into low, medium, and high FBG groups (FBG < 7.5 mmol/L, 7.5 mmol/L ≤ FBG < 8.73 mmol/L, and FBG ≥ 8.73 mmol/L, respectively). The baseline characteristics and insulin secretory index were compared among the three groups. Each group was divided into three subgroups according to BMI: lean (BMI < 24 kg/m^2^), overweight (24 ≤ BMI < 28 kg/m^2^), and obese (BMI ≥ 28 kg/m^2^) subgroups.

### 2.4. Statistical Methods

The study used SPSS version 21.0 for statistical analysis. Continuous variables with normal distributions were expressed as mean ± standard deviation (SD). The Student *t*-test and one-way analysis of variance (ANOVA) were applied to analyze the differences between the groups. Continuous variables that have abnormal distribution were given as median with a range of upper and lower quartiles and analyzed using a nonparametric test. Discontinuous variables were expressed as percentage and analyzed using the chi-square test. In addition, logistic regression was performed for correlation analysis. Statistical significance was defined as *p* < 0.05.

## 3. Results

### 3.1. Characteristics and Insulin Secretion Function among Different FBG Groups

**Figure 1:**
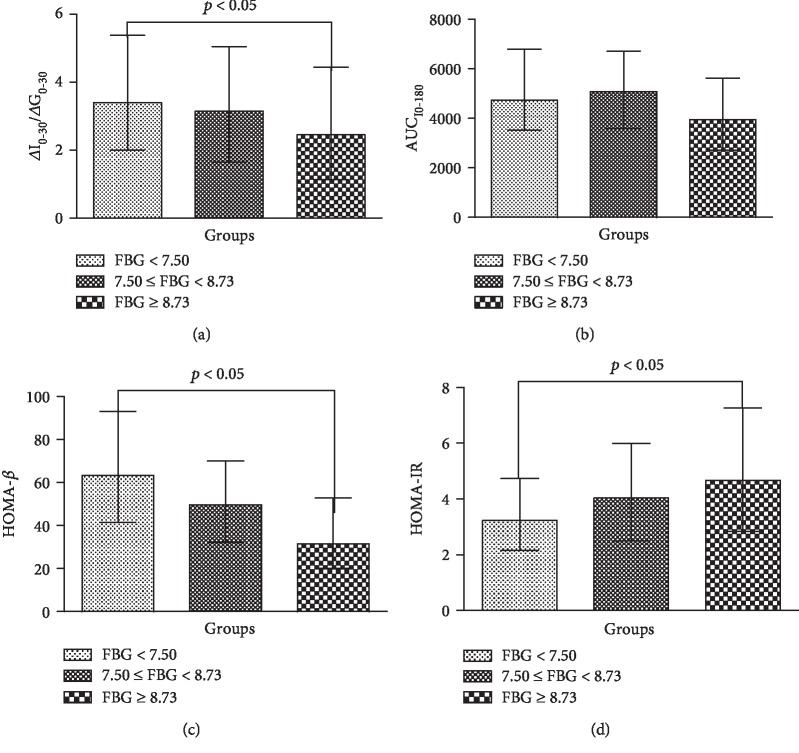
Comparison of *β*-cell function including (a) *Δ*I_0-30_/*Δ*G_0-30_, (b) AUC_I0-180_, (c) HOMA-*β*, and (d) HOMA-IR among different FBG level groups. HOMA-IR: homeostasis model assessment of insulin resistance; HOMA-*β*: homeostasis model assessment of *β*-cell function; AUC_I0-180_: area under the insulin curve.

Baseline characteristics and insulin secretion function of the three different FBG groups have been summarized in Table 1 and Figure 1. A significant increase of TG (1.77 (1.23-2.54) vs. 1.80 (1.29-2.62) vs. 2.12 (1.36-2.91) mmol/L) and HOMA-IR (3.23 (2.16-4.74) vs. 4.04 (2.50-6.00) vs. 4.68 (2.84-7.27)) and a significant decrease of EISI (3.39 (2.01-5.39) vs. 3.14 (1.65-5.05) vs. 2.45 (1.13-4.44)) and HOMA-*β* (63.25 (41.49-92.87) vs. 49.44 (32.12-69.97) vs. 31.56 (20.15-52.83)) were shown among the low, medium, and high FBG groups. In the medium FBG group, AUC_I0-180_ was higher than that in the low and high FBG groups (5084.63 (3574.80-6710.10) vs. 4733.4 (3522.1-6792.1) vs. 3944.10 (2720.33-5618.78)), and the differences were significant (*p* < 0.05).

Multiple logistic regression analysis was performed to analyze factors that were associated with the early phase of insulin secretion (Table 2). EISI was used as dependent variable and BMI, age, sex, DBP, TG, and FBG were used as independent variables. BMI ≥ 28 kg/m^2^ (odds ratio (OR): 1.943; 95% confidence interval (CI): 1.207-3.128) and FBG < 7.50 mmol/L (OR: 1.868; 95% CI: 1.274-2.739) had a positive correlation with EISI.

### 3.2. Characteristics and Insulin Secretion Function among Different BMI Subgroups in Different FBG Groups

**Figure 2:**
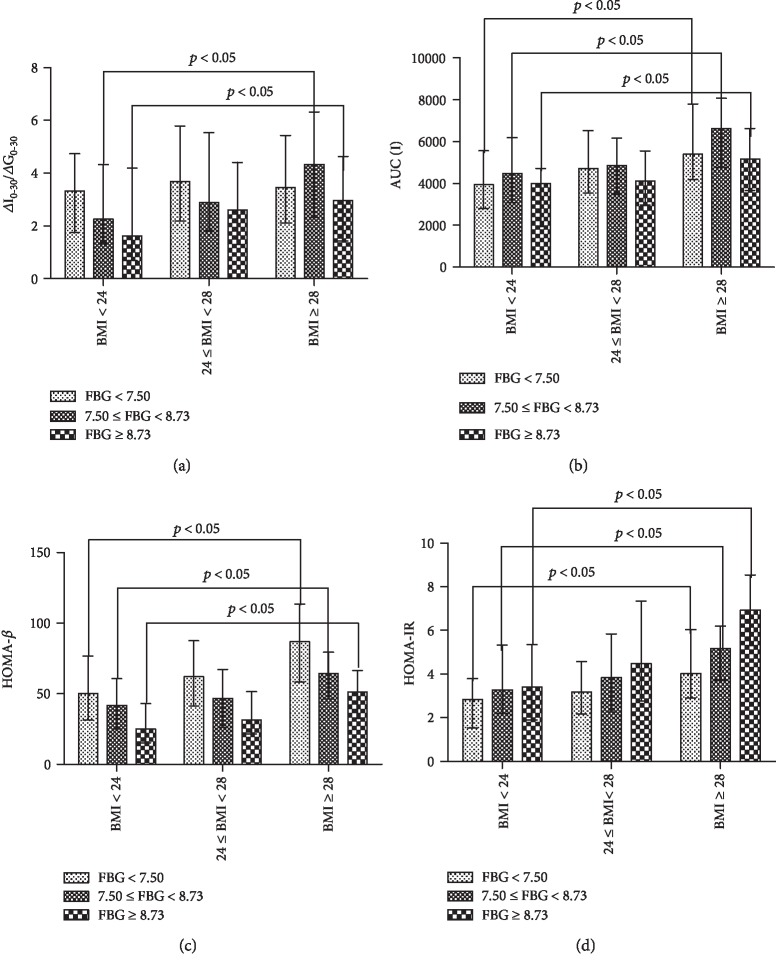
Comparison of *β*-cell function including (a) *Δ*I_0-30_/*Δ*G_0-30_, (b) AUC_I0-180_, (c) HOMA-*β*, and (d) HOMA-IR among different BMI subgroups in different FBG level groups. HOMA-IR: homeostasis model assessment of insulin resistance; HOMA-*β*: homeostasis model assessment of *β*-cell function; AUC_I0-180_: area under the insulin curve.

In the low FBG group, baseline characteristics and insulin secretion functions were compared among different BMIs (Table 3 and Figure 2). A significant increase in DBP (75.8 ± 8.5 vs. 78.7 ± 8.3 vs. 81.6 ± 10.0 mm Hg), AUC_I0-180_ (3936.75 (2815.13-5564.33) vs. 4714.95 (3540.45-6529.05) vs. 5394.00 (4194.90-7799.10)), HOMA-*β* (50.00 (31.27-76.72) vs. 62.02 (41.34-87.62) vs. 86.81 (58.42-113.41)), and HOMA-IR (2.83 (1.54-3.80) vs. 3.18 (2.18-4.57) vs. 4.02 (2.91-6.04)) was noted among lean, overweight, and obese patients. However, EISI had no significant difference with increase in BMI. Logistic regression showed that FBG was negatively correlated with EISI (OR: 0.038; 95% CI: 0.330-0.970), whereas BMI had no obvious correlation with EISI (Table 4).

For the medium FBG group, the comparison of baseline and insulin secretion functions is summarized in Table 5 and Figure 2. As BMI increased, not only DBP (76.7 ± 7.6 vs. 80.2 ± 7.2 vs. 81.0 ± 9.9 mm Hg), TC (5.15 ± 1.24 vs. 5.19 ± 1.18 vs. 5.46 ± 1.19 mmol/L), and TG (1.54 (1.26-2.51) vs. 1.75 (1.21-2.42) vs. 2.16 (1.53-3.12) mmol/L) but also EISI (2.26 (1.32-4.33) vs. 2.88 (1.81-4.54) vs. 4.33 (2.31-6.32)), HOMA-*β* (41.77 (24.91-60.66) vs. 46.80 (26.22-67.30) vs. 64.24 (46.36-79.44)), HOMA-IR (3.27 (2.20-5.32) vs. 3.84 (2.26-5.84) vs. 5.18 (3.73-6.20)), and AUC_I0-180_ (4479.00 (3085.20-6195.90) vs. 4856.25 (3506.70-6165.45) vs. 6631.35 (4770.68-8067.00)) increased significantly (*p* < 0.05). Logistic regression showed that BMI ≥ 28 kg/m^2^ (OR: 0.566; 95% CI: 0.330-0.970) had a correlation with EISI independently, whereas FBG had no obvious correlation with EISI (Table 6).

In the high FBG group, a significant increase in DBP (78.0 ± 7.0 vs. 79.4 ± 9.0 vs. 83.7 ± 7.1 mm Hg), HOMA-*β* (25.07 (15.33-42.92) vs. 31.48 (21.60-51.42) vs. 51.32 (32.56-66.47)), HOMA-IR (3.41 (1.89-5.35) vs. 4.48 (2.76-7.35) vs. 6.92 (5.31-8.54)), and AUC_I0-180_ (3994.95 (1945.58-4722.64) vs. 4114.20 (2972.03-5557.76) vs. 5171.85 (3669.45-6633.30)) and a significant decrease in HDL (1.30 ± 0.28 vs. 1.22 ± 0.33 vs. 1.18 ± 0.24 mmol/L) were found among lean, overweight, and obese groups (Table 7 and Figure 2). EISI had no significant difference among the groups. Logistic regression analysis was used to analyze factors associated with early-phase insulin secretion. After adjusting for DBP, TG, sex, and age, there was a significant negative correlation between FBG and EISI (OR: 0.664; 95% CI: 0.482-0.914), whereas there was no obvious correlation between BMI and EISI (Table 8).

## 4. Discussion

In the present study, during the progression of baseline FBG, *β*-cell function decreased and insulin resistance increased significantly in newly diagnosed T2DM patients. It suggested that in patients who already have T2DM, part of the compensatory capacity of islet secretion function is lost, and with the increase of FBG, the compensatory capacity becomes worse. Both insulin secretion defect and insulin resistance are major mechanisms of T2DM. It is well known that *β*-cell dysfunction has been present as hyperglycemia to exist in T2DM. The Belfast Diet study demonstrated that within 6 years after the diagnosis of 67 newly diagnosed type 2 diabetic patients treated with diet control, the decrease in *β*-cell function contributed more to T2DM. This study was conservative in treatment but found the relationship between insulin resistance and islet *β*-cell dysfunction in the natural progression of diabetes mellitus [6]. Similarly, the United Kingdom Prospective Diabetes Study (UKPDS) showed that the progressive nature of diabetes is that the function of *β*-cells continues to decrease while insulin sensitivity remains unchanged in people recently diagnosed with T2DM [7, 8]. The decrease of first-phase response of insulin release is a critical index related directly to the development of diabetes mellitus and was found consistently absent when fasting plasma glucose was over 115 mg/dL [9, 10]. In addition to HOMA-*β*, this study also used EISI as an important index to evaluate *β*-cell function.

The current study found that HOMA-*β* and EISI decreased as FBG increased in patients with T2DM. The study showed a strong inverse relationship between *β*-cell dysfunction (HOMA-*β* and EISI) and FBG. A previous study among normal glucose tolerance (NGT), impaired glucose tolerance (IGT), and type 2 diabetes mellitus (T2DM) patients showed that the EISI of the T2DM group was significantly lower than that of the NGT and IGT groups [11]. And the secretion of glucagon-like peptide-l was positively correlated with EISI [11]. Jensen et al. also found that decreasing glucose tolerance was associated with decreasing EISI, and it was also identified when adjusting this measure for insulin sensitivity (EISI/HOMA-IR) in four ethnic groups (African-American, Asian-American, Caucasian, and Hispanic-American) [12]. Similar with the current study, another cross-sectional study in Korea analyzed by Kim et al. showed that EISI decreased significantly with increasing quartiles of HbA1C values, and the higher HbA1C was associated with impaired early-phase insulin secretion [13]. Whereas different from previous studies, our study examined subjects with newly diagnosed T2DM, and we assessed the potential difference of *β*-cell dysfunction during the period from fasting glucose processing.

The HbA1c levels were found significantly increased as FBG progressed in the current study. HbA1c is an indicator of average blood glucose for diabetic or nondiabetic patients. Similar with the result of our study, FBG and HbA1c were found to have a significant positive correlation in diabetic subjects [14]. However, HbA1c is not correlated with FBG in subjects without diabetes [15]. Some studies have shown that with the increase of HbA1c, the contribution of postprandial blood glucose excurses, while the contribution of FBG increses [16–18].

In our study, a significant increase of AUC(I), HOMA-*β*, and HOMA-IR was found among lean, overweight, and obese patients at any level of the FBG group. Logistic analyses in the present study found that high EISI was associated with higher BMI. Obesity is one of the important factors for insulin resistance, and insulin resistance is the pathogenic basis of obesity and T2DM [19, 20]. Many studies have shown that adipose tissue of obese subjects results in insulin resistance and other metabolic dysfunction by releasing nonesterified fatty acids, glycerol, leptin, adiponectin, and proinflammatory cytokines, which interfere with the insulin signal transduction pathway and downregulate gene expression of insulin receptor substrates through different mechanisms [21–24]. A study of Japanese [25] subjects with prediabetes indicated that insulin sensitivity (Matsuda index) was lower and EISI was significantly higher in obese subjects than nonobese subjects in each decile of 2-hour glucose level by OGTT. However, there was no significant difference in disposition index (EISI/HOMA-IR) between the nonobese and obese groups. A study in Korea with new-onset T2DM patients also reported that higher BMI was associated with higher early insulin response but associated with a lower disposition index [26]. In this study, we analyzed the function of insulin secretion in obese, overweight, and lean patients with newly diagnosed T2DM at different levels of FBG. The results suggested that the compensatory insulin secretion function in obese patients increased compared with nonobese patients with T2DM.

In the general subjects, we found that both FBG and BMI were independently associated with early insulin secretion. We further studied the factors that correlated to EISI of patients with different FBG levels and found that the factors independently related to EISI were different in specific baseline FBG groups. In patients with FBG ≥ 8.73 mmol/L and FBG < 7.50 mmol/L, FBG was the main factor associated with first-phase *β*-cell secretion function, whereas BMI was not the main factor affecting it. Increasing BMI has a significant positive effect on early-phase insulin secretion in patients with fasting blood glucose from 7.50 to 8.73 mmol/L, whereas FBG level has no significant correlation with early insulin secretion. Our findings indicated that at particular fasting glucose levels, obesity has a positive effect on pancreatic islet dysfunction.

Large numbers of subjects were needed to evaluate the relationship between *β*-cell function and increasing FBG and BMI to confirm our result. The present study was a cross-sectional study. Thus, follow-up studies are required to further evaluate the changes of *β*-cell function during the development of diabetes in such patients.

## 5. Conclusions

In conclusion, during the progression of baseline FBG, *β*-cell dysfunction and insulin resistance worsened in newly diagnosed T2DM patients. As FBG increased, increased BMI had a positive influence on *β*-cell dysfunction. The independent factors that correlated with EISI were different with the increasing of baseline FBG.

## Figures and Tables

**Table 1:** Basic characteristics among different FBG groups.

	<7.50 *n* = 231	7.50 ≤ FBG < 8.73 *n* = 209	≥8.73 *n* = 221	*p*
Male, *n* (%)	131 (56.7)	125 (59.8)	133 (60.2)	0.713
Age (y)	50.1 ± 9.2	52.4 ± 9.0	49.3 ± 9.4	0.368
Average timing of diabetes diagnosed (y)	0.13 (0.09-0.21)	0.13 (0.09-0.24)	0.15 (0.09-0.35)	0.054
BMI (kg/m^2^)	25.75 ± 2.60	25.96 ± 2.49	25.42 ± 2.52	0.179
Waist circumference (cm)	89.11 ± 8.53	90.75 ± 8.06	88.51 ± 8.30	0.460
SBP (mm Hg)	123.0 ± 12.7	124.3 ± 12.3	124.0 ± 13.1	0.381
DBP (mm Hg)	78.6 ± 8.9	79.6 ± 8.2	79.8 ± 8.3	0.136
TC (mmol/L)	5.24 ± 1.20	5.22 ± 1.05	5.40 ± 1.15	0.148
TG (mmol/L)	1.77 (1.23-2.54)	1.80 (1.29-2.62)	2.12 (1.36-2.91)	0.031
LDL-C (mmol/L)	3.08 ± 0.93	3.06 ± 0.87	3.07 ± 0.89	0.878
HDL-C (mmol/L)	1.24 ± 0.32	1.22 ± 0.28	1.24 ± 0.30	0.885
HbA1c (%)	7.05 ± 1.03	7.36 ± 1.00	8.04 ± 1.20^∗^	0.000

^∗^Significant statistical differences compared to the low FBG group (*p* values < 0.05). BMI: body mass index; SBP: systolic blood pressure; DBP: diastolic blood pressure; TC: total cholesterol; LDL-C: low-density lipoprotein cholesterol; HDL-C: high-density lipoprotein cholesterol; TG: triglyceride; PBG: 2 h blood glucose of OGTT; HbA1c: hemoglobin A1c.

**Table 2:** Logistic regression analyzed factors associated with *Δ*I_30_/*Δ*G_30_.

	*p* value	OR	95% CI
BMI ≥ 28	0.006	1.943	1.207-3.128
24 ≤ BMI < 28	0.168	1.308	0.893-1.917
BMI < 24			
FBG < 7.50	0.001	1.868	1.274-2.739
7.50 ≤ FBG < 8.73	0.083	1.415	0.956-2.096
FBG ≥ 8.73			

Compared to ΔI_30_/ΔG_30_ < 3.07. Variables included in the model were male, age, BMI, fasting blood glucose, diastolic blood pressure, and triglyceride. BMI: body mass index; TG triglyceride; FBG: fasting blood glucose; SBP: systolic blood pressure.

**Table 3:** Basic characteristics among different BMI subgroups in the low FBG group.

	<24 *n* = 58	24 ≤ BMI < 28 *n* = 122	≥28 kg/m^2^ *n* = 51	*p*
Male, *n* (%)	26 (44.8)	69 (56.6)	36 (70.6)	0.026
Age (y)	51.6 ± 10.0	50.5 ± 8.8	47.5 ± 8.8^∗^	0.171
Average timing of diabetes diagnosed (y)	0.12 (0.09-0.22)	0.12 (0.10-0.23)	0.10 (0.08-0.18)	0.212
Waist circumference (cm)	81.26 ± 6.95	89.47 ± 6.24	97.17 ± 6.91^∗^	0.000
SBP (mm Hg)	121.5 ± 13.0	123.4 ± 11.9	123.5 ± 14.0	0.860
DBP (mm Hg)	75.8 ± 8.5	78.7 ± 8.3	81.6 ± 10.0^∗^	0.008
TC (mmol/L)	5.15 ± 1.24	5.19 ± 1.18	5.46 ± 1.19	0.165
TG (mmol/L)	1.54 (1.17-2.25)	1.80 (1.29-2.49)	2.05 (1.16-3.50)	0.117
LDL-C (mmol/L)	2.98 ± 1.07	3.09 ± 0.91	3.17 ± 0.83	0.095
HDL-C (mmol/L)	1.31 ± 0.46	1.23 ± 0.24	1.20 ± 0.30	0.213
FBG (mmol/L)	6.90 ± 0.54	6.87 ± 0.54	6.82 ± 0.55	0.432
HbA1c (%)	6.99 ± 1.02	7.09 ± 1.13	7.01 ± 0.73	0.781

^∗^Significant statistical differences compared to BMI < 24 group (*p* values < 0.05). BMI: body mass index; SBP: systolic blood pressure; DBP: diastolic blood pressure; TC: total cholesterol; LDL-C: low-density lipoprotein cholesterol; HDL-C: high-density lipoprotein cholesterol; TG: triglyceride; FBG: fasting blood glucose; HbA1c: hemoglobin A1c.

**Table 4:** Logistic regression analyzed factors associated with *Δ*I_30_/*Δ*G_30_ in the low FBG group.

	*p* value	OR	95% CI
BMI ≥ 28	0.978	1.011	0.445-2.297
24 ≤ BMI < 28	0.605	1.190	0.616-2.298
BMI < 24			
FBG	0.038	0.566	0.330-0.970

Compared to ΔI_30_/ΔG_30_ < 3.07. Variables included in the model were male, age, BMI, fasting blood glucose, diastolic blood pressure, and triglyceride. BMI: body mass index; TG triglyceride; FBG: fasting blood glucose; *p* values < 0.05.

**Table 5:** Basic characteristics among different BMI subgroups in the medium FBG group.

	<24 *n* = 50	24 ≤ BMI < 28 *n* = 105	≥28 kg/m^2^ *n* = 54	*p*
Male, *n* (%)	31 (62.0)	62 (59.0)	32 (59.3)	0.026
Age (y)	53.3 ± 9.8	53.4 ± 8.6	49.8 ± 8.3	0.040
Average timing of diabetes diagnosed (y)	0.15 (0.10-0.38)	0.13 (0.88-0.23)	0.11 (0.09-0.19)	0.153
Waist circumference (cm)	83.33 ± 6.60	90.79 ± 6.26	97.54 ± 6.22^∗^	0.000
SBP (mm Hg)	121.0 ± 12.3	126.4 ± 13.0	123.5 ± 14.1	0.362
DBP (mm Hg)	76.7 ± 7.6	80.2 ± 7.2	81.0 ± 9.9^∗^	0.008
TC (mmol/L)	5.15 ± 1.24	5.19 ± 1.18	5.46 ± 1.19^∗^	0.034
TG (mmol/L)	1.54 (1.26-2.51)	1.75 (1.21-2.42)	2.16 (1.53-3.12)	0.014
LDL-C (mmol/L)	2.94 ± 0.87	3.09 ± 0.89	3.12 ± 0.85	0.294
HDL-C (mmol/L)	1.21 ± 0.25	1.27 ± 0.30	1.18 ± 0.35	0.520
FBG (mmol/L)	8.02 ± 0.37	8.12 ± 0.36	7.96 ± 0.32	0.380
HbA1c (%)	7.34 ± 1.23	7.43 ± 1.00	7.24 ± 0.77	0.597

^∗^Significant statistical differences compared to BMI < 24 group (*p* values < 0.05). BMI: body mass index; SBP: systolic blood pressure; DBP: diastolic blood pressure; TC: total cholesterol; LDL-C: low-density lipoprotein cholesterol; HDL-C: high-density lipoprotein cholesterol; TG: triglyceride; FBG: fasting blood glucose; HbA1c: hemoglobin A1c.

**Table 6:** Logistic regression analyzed factors associated with *Δ*I_30_/*Δ*G_30_ in the medium FBG group.

	*p* value	OR	95% CI
BMI ≥ 28	0.014	2.848	1.234-6.571
24 ≤ BMI < 28	0.385	1.372	0.672-2.802
BMI < 24			
FBG	0.082	0.481	0.211-1.098

Compared to ΔI_30_/ΔG_30_ < 3.07. Variables included in the model were male, age, BMI, fasting blood glucose, diastolic blood pressure, and triglyceride. BMI: body mass index; FBG: fasting blood glucose; *p* values < 0.05.

**Table 7:** Basic characteristics among different BMI subgroups in the high FBG group.

	<24 *n* = 68	24 ≤ BMI < 28 *n* = 110	≥28 kg/m^2^ *n* = 43	*p*
Male, *n* (%)	31 (45.6)	72 (65.5)	30 (69.8)	0.026
Age (y)	50.4 ± 9.8	49.26 ± 9.8	47.7 ± 7.6	0.141
Average timing of diabetes diagnosed (y)	0.16 (0.09-0.27)	0.15 (0.09-0.33)	0.15 (0.09-0.38)	0.981
Waist circumference (cm)	81.33 ± 6.96	89.80 ± 5.85	96.54 ± 6.44^∗^	0.000
SBP (mm Hg)	124.4 ± 12.8	122.8 ± 14.0	126.6 ± 10.7	0.529
DBP (mm Hg)	78.0 ± 7.0	79.4 ± 9.0	83.7 ± 7.1^∗^	0.001
TC (mmol/L)	5.41 ± 1.15	5.47 ± 1.27	5.19 ± 0.79	0.401
TG (mmol/L)	1.80 (1.16-2.66)	2.18 (1.38-2.91)	2.39 (1.152-3.13)^∗^	0.091
LDL-C (mmol/L)	3.13 ± 0.90	3.07 ± 0.95	2.96 ± 0.68	0.337
HDL-C (mmol/L)	1.30 ± 0.28	1.22 ± 0.33	1.18 ± 0.24^∗^	0.032
FBG (mmol/L)	10.04 ± 1.22	9.91 ± 1.00	9.93 ± 0.81	0.527
HbA1c (%)	8.04 ± 1.24	8.07 ± 1.14	7.94 ± 1.30	0.781

^∗^Significant statistical differences compared to BMI < 24 group (*p* values < 0.05). BMI: body mass index; SBP: systolic blood pressure; DBP: diastolic blood pressure; TC: total cholesterol; LDL-C: low-density lipoprotein cholesterol; HDL-C: high-density lipoprotein cholesterol; TG: triglyceride; FBG: fasting blood glucose; HbA1c: hemoglobin A1c.

**Table 8:** Logistic regression analyzed factors associated with *Δ*I_30_/*Δ*G_30_ in the high FBG group.

	*p* value	OR	95% CI
BMI ≥ 28	0.222	1.674	0.732-3.826
24 ≤ BMI < 28	0.492	1.258	0.654-2.421
BMI < 24			
FBG	0.012	0.664	0.482-0.914

Compared to ΔI_30_/ΔG_30_ < 3.07. Variables included in the model were male, age, BMI, fasting blood glucose, diastolic blood pressure, and triglyceride. BMI: body mass index; FBG: fasting blood glucose; *p* values < 0.05.

## Data Availability

The data used to support the findings of this study are available from the corresponding author upon request.
